# Quality of Life in Patients with Gluten Neuropathy: A Case-Controlled Study

**DOI:** 10.3390/nu10060662

**Published:** 2018-05-23

**Authors:** Panagiotis Zis, Ptolemaios Georgios Sarrigiannis, Dasappaiah Ganesh Rao, Marios Hadjivassiliou

**Affiliations:** Academic Department of Neurosciences, Sheffield Teaching Hospitals NHS Foundation Trust, Sheffield S10 2JF, South Yorkshire, UK; Ptolemaios.Sarrigiannis@sth.nhs.uk (P.G.S.); Ganesh.Rao@sth.nhs.uk (D.G.R.); m.hadjivassiliou@sheffield.ac.uk (M.H.)

**Keywords:** gluten neuropathy, coeliac disease, gluten free diet, quality of life, male

## Abstract

Background: Gluten neuropathy (GN) is defined as an otherwise idiopathic peripheral neuropathy in the presence of serological evidence of gluten sensitivity (positive native gliadin antibodies and/or transglutaminase or endomysium antibodies). We aimed to compare the quality of life (QoL) of GN patients with that of control subjects and to investigate the effects of a gluten-free diet (GFD) on the QoL. Methods: All consecutive patients with GN attending a specialist neuropathy clinic were invited to participate. The Overall Neuropathy Limitations Scale (ONLS) was used to assess the severity of the neuropathy. The 36-Item Short Form Survey (SF-36) questionnaire was used to measure participants’ QoL. A strict GFD was defined as effectively being able to eliminate all circulating gluten sensitivity-related antibodies. Results: Fifty-three patients with GN and 53 age- and gender-matched controls were recruited. Compared to controls, GN patients showed significantly worse scores in the physical functioning, role limitations due to physical health, energy/fatigue, and general health subdomains of the SF-36. After adjusting for age, gender, and disease severity, being on a strict GFD correlated with better SF-36 scores in the pain domain of the SF-36 (beta 0.317, *p* = 0.019) and in the overall health change domain of the SF-36 (beta 0.306, *p* = 0.017). Conclusion: In GN patients, physical dysfunctioning is the major determinant of poor QoL compared to controls. Routine checking of the elimination of gluten sensitivity-related antibodies that results from a strict GFD should be encouraged, as such elimination ameliorates the overall pain and health scores, indicating a better QoL.

## 1. Introduction

Gluten neuropathy (GN) is one of the commonest neurological manifestations of gluten sensitivity [1] and it is defined as an otherwise idiopathic peripheral neuropathy (PN) [2] in the presence of serological evidence of gluten sensitivity (positive native gliadin antibodies, and/or transglutaminase or endomysium antibodies) [1,3]. Some patients with GN have evidence of enteropathy revealed by duodenal biopsy and are diagnosed with coeliac disease (CD), whereas the majority of patients with GN do not have enteropathy.

The main type of gluten neuropathy is symmetrical sensorimotor axonal peripheral neuropathy [1], however sensory ganglionopathy (SG) and, rarely, mononeuritis multiplex (MMX) may also occur [1,4,5]. A gluten-free diet (GFD) has been shown to be effective in treating GN, irrespective of the presence or not of enteropathy [6].

As in all axonal neuropathies, symptoms can be divided into sensory and motor. Incoordination and gait disturbance are symptoms usually attributed to damage of the sensory nerves (sensory ataxia) [2]. Other sensory symptoms include tingling, pins and needles, numbness, tightness, burning, and pain. Motor symptoms include muscle cramps, stiffness, weakness, and wasting [2]. The clinical diagnosis is confirmed with nerve conduction studies.

Robust epidemiological data on the prevalence of GN neuropathy are lacking, however it is known that PN of any etiology can affect between 2.4% and 8% of the general population [2] and that CD is associated with a 2.5-fold increased risk of later neuropathy [5]. 

As in all chronic diseases, patients with GN are expected to have an impaired quality of life (QoL) for reasons that are either directly or indirectly related to the disease. It has been shown that patients with advanced peripheral neuropathy, for example, show worse scores in questionnaires measuring QoL when compared to less impaired patients [7]. Such impairment, refers not only to motor symptoms (i.e., weakness) but also to sensory symptoms, in particular pain [8,9,10,11,12,13]. Indirectly, however, GN might cause an additional burden by having to adhere to a strict GFD. It has been shown that the degree of difficulty in adhering to a GFD is associated with reductions in patient wellbeing and psychological distress that the dietician is critically placed to address [14].

The purpose of this study was twofold. We wanted to compare the QoL of GN patients with that of controls (subjects without peripheral neuropathy or gluten sensitivity) and to investigate the effect of being on a GFD on the QoL in these patients.

## 2. Methods

### 2.1. Procedure and Participants

This was a cross-sectional study conducted at the Sheffield Institute of Gluten-Related Diseases (SIGReD). Patients were recruited during their regular visits to the gluten/neurology clinic based at the Royal Hallamshire Hospital, Sheffield, UK. Individuals (i.e., carers or relatives) without a diagnosis of PN or risk factors for developing PN participated as controls.

To be enrolled, the patients had to meet the following inclusion criteria: (1) diagnosis of PN, as confirmed by nerve conduction studies, (2) serological evidence of gluten sensitivity (positive for native gliadin IgG and/or IgA antibodies with or without positivity for endomysial and transgultaminase antibodies) at diagnosis prior to commencing a gluten-free diet, (3) absence of other risk factors for developing PN (i.e., diabetes, vitamin deficiencies, exposure to neurotoxic agents) (4) age equal to or greater than 18 years, (5) able to provide a written informed consent.

The study protocol was approved by the local ethics committee.

### 2.2. Measures

The demographic characteristics included age and gender. All patients went through extensive investigations for possible causes of PN [2]. Patients with a family history of neuropathy were excluded.

The type of neuropathy (sensorimotor length-dependent PN, sensory ganglionopathy [15,16], mononeuritis multiplex) for all patients was determined on the basis of nerve conduction studies, which were performed by the same clinician on the day of the recruitment.

All patients were referred for an endoscopy and duodenal biopsy to establish the presence of enteropathy. All biopsies were histologically assessed by an experienced pathologist for evidence of enteropathy (triad of villous atrophy, crypt hyperplasia, and increase in intraepithelial lymphocytes).

The severity of neuropathy was assessed by the Overall Neuropathy Limitations Scale (ONLS), which is a validated scale that measures limitations in the everyday activities of the upper and lower limbs [17].

Biagi score was used to document adherence to a gluten-free diet [18]. Patients with a Biagi score of equal to or above 3 were considered as being on a gluten-free diet. Furthermore, patients with negative serology at the time of recruitment were considered to be on a strict gluten-free diet.

The 36-Item Short Form Survey (SF-36), a self-reported measure of health status and quality of life [19], was used to determine patient health-related quality of life. SF-36 covers nine health and QoL domains. These domains include physical functioning, role limitations due to physical health, role limitations due to emotional problems, energy/fatigue, emotional well-being, social functioning, pain, general health and health change. Each item is measured using a Likert-type scale. The scores were converted and analysed according to the marking guidelines for the SF-36, such that higher scores (out of a total of 100 for each domain) constitute better health-related quality of life in this domain.

### 2.3. Statistical Analyses

A database was developed using the Statistical Package for Social Science (version 23.0 for Mac; SPSS). Frequencies and descriptive statistics were examined for each variable. Comparisons between patients on a gluten-free diet and patients not on a gluten-free diet were made using Student’s t-tests for normally distributed continuous data, Mann–Whitney’s U test for non-normally distributed, and chi-square test or Fischer’s exact test for categorical data.

Where differences with a p value lower than 0.10 were found, these variables were entered into linear regression models, with the QoL domain score being the dependent variable. All accuracy and generalization assumptions for the model were checked.

The level of statistical significance was set at the 0.05 level.

## 3. Results

### 3.1. Study Population

Fifty-three patients with GN were recruited (73.6% male, mean age 68.2 ± 9.3 years) and were age- and gender-matched with 53 control subjects without a history of peripheral neuropathy or gluten sensitivity. Thirty-six patients (67.9%) had a symmetrical length-dependent sensorimotor axonal PN, 16 (30.2%) had a sensory ganglionopathy, and 1 patient had mononeuritis multiplex (1.9%). The mean disease duration was 12.6 ± 9.5 years (ranging from 0 to 37 years), suggesting that the GN is a late extra-intestinal manifestation of serologically confirmed gluten sensitivity (occurring usually in the sixth decade of life). Overall ONLS scores ranged from 1 to 7 (mean 3.1 ± 1.8).

Not all of the patients agreed to a duodenal biopsy. Of 32 patients who underwent duodenal biopsy, nine (28.1%) had enteropathy (eight coeliac disease—Marsh type 3, one increased intraepithelial lymphocytes—Marsh type 2).

### 3.2. QoL Compared to Controls

Table 1 summarizes the scores in all SF-36 subdomains in patients with GN and in controls. Patients with GN showed significantly worse scores compared to controls in the following quality of life modalities: physical functioning (*p* < 0.001), role limitations due to physical health (*p* < 0.001), energy/fatigue (*p* = 0.045), and general health (*p* = 0.029). A trend of statistical significance (*p* = 0.094) was observed in the pain modality of the SF-36 questionnaire.

### 3.3. The Role of GFD (Self-Reported) on QoL

On the basis of the self-reported adherence to GFD (Biagi score equal to or greater than 3), 31 patients (58.5%) were on a GFD. Table 2 summarizes the demographic, clinical, and quality-of-life-related characteristics of patients with GN reporting being on GFD versus patients with GN reporting being on a gluten-containing diet. The two groups did not differ significantly in age, gender, disease duration, neuropathy severity, or neuropathy type. A trend of statistical significance (*p* = 0.094) was observed on the overall health change subdomain of the SF-36.

After adjusting for age, gender, and disease severity, being on GFD (self-reported) was positively correlated with better SF-36 scores on the overall health change domain of the SF-36 (beta 0.258, *p* = 0.047).

### 3.4. The Role of Strict GFD on QoL

Twenty-two patients managed to eliminate antigliadin, endomysial, and transglutaminase antibodies by adopting the GFD. This population, which accounted for 41.5% of the total GN study group and 71% of those GN patients self-reporting as being on a GFD, was considered to be on a strict GFD.

Table 3 summarizes the demographic, clinical, and quality-of-life-related characteristics of patients with GN being on a strict GFD versus patients with GN not on a strict GFD. The two groups did not differ significantly in age, gender, disease duration, neuropathy severity, or neuropathy type. Patients on a strict GFD had significantly higher scores (indicating better QoL) on the pain sub-domain of the SF-36 (*p* = 0.03). A trend of statistical significance (*p* = 0.066) was also observed on the overall health change subdomain of the SF-36.

After adjusting for age, gender, and disease severity, being on a strict GFD (serologically proven) was positive correlated with better SF-36 scores on the pain domain of the SF-36 (beta 0.317, *p* = 0.019) and the overall health change domain of the SF-36 (beta 0.306, *p* = 0.017).

## 4. Discussion

This case-controlled study demonstrates that patients with GN have significantly worse QoL compared to age- and gender-matched controls on the basis of the SF-36 modalities of physical functioning, role limitations due to physical health, energy/fatigue, and general health. This finding adds to the existing literature that the main impact of peripheral neuropathy on patients’ QoL is on the modalities affecting their dysfunctioning (i.e., impaired activities of daily living) [7]. To our knowledge, this is the first study investigating the QoL of patients with GN.

Another novelty of the current study is the examination of the role of GFD on QoL. For this, we conducted two separate analyses, one based on the patients' reports and the other based on the evidence of the elimination of all gluten sensitivity-related antibodies.

In their study, Lee et al. found that patients with coeliac disease reported that GFD had a negative impact on their quality of life, as it restricted their social activities such as travelling or dining out [20]. In our study, patients on a GFD had higher scores in the sub-domains of SF-36 (Table 2), though not statistically significant. There was, however, a trend for a statistically significant difference in the scores of the overall health change subdomain of the SF-36. There are possible explanations for such disparity. Firstly, awareness of gluten sensitivity and coeliac disease has increased over the last decade with improved availability and a better range of gluten-free products. Furthermore, dining out or travelling is much easier, as many restaurants and hotels do have gluten-free menus. Secondly, the heterogeneity of the study populations may have affected the results, as Lee et al. included patients with different gender distribution (female predominance), different age (younger), and a different clinical picture (coeliac disease with gastrointestinal symptoms), whereas our cohort of patients presented a neuropathy.

When comparing patients who strictly adhered to the GFD (as evidenced by the elimination of all circulating gluten sensitivity-related antibodies) with patients either not being on the GFD or not being strictly on the GFD, we showed that the former had significantly better scores in the pain and the overall health change domain. This is in keeping with the current literature, which shows that patients with GN benefit from a GFD with evidence of improvement of the neuropathy usually after one year on strict GFD, associated with the elimination of gluten sensitivity-related antibodies [6,20]. Moreover, this finding highlights the importance of serological monitoring in an attempt to improve adherence to a GFD. Interestingly, in our cohort, 29% of patients on the GFD still had positive serology.

Our study population comprised predominantly males (male to female ratio 3:1). As we recruited patients in succession, this might indicate that GN is commoner in males, which is in contrast with what Thawani et al. reported in their epidemiological study where they assessed the risk of neuropathy among patients with CD and found no difference in the risk of developing neuropathy between the two genders [5]. This difference in findings might be due to the fact that the majority of patients with GN do not have enteropathy and, therefore, the male predominance possibly reflects this. Our observation, however, is important, as male patients perceive illness and quality of life differently, and adherence to a GFD has a different emotional impact in males compared to females [21].

Our results should be interpreted with some caution, given the limitations of our design. This is a cross-sectional study based on patients attending a specialized clinic, and the results may not be generalizable to other settings. Furthermore, our cohort included patients with large fiber axonal peripheral neuropathies only. Pure small fiber neuropathy associated with gluten sensitivity is another area that merits further consideration, as it is a particularly painful condition and therefore can affect patients’ QoL.

In conclusion, in patients with GN, physical dysfunctioning is the major determinant of QoL compared to control subjects. Contrary to previous observations in patients with classical CD, being on a GFD does not have a negative effect on social functioning in patients with GN. Clinicians are advised to regularly monitor the adherence to the GFD diet by serological testing for gluten sensitivity-related antibodies, because a strict GFD ameliorates the overall pain and health change scores, indicating better QoL.

## Figures and Tables

**Table 1:** Demopgraphics and quality of life (QoL) parameters of patients with gluten neuropathy (GN).

	GN (*n* = 53)	Controls (*n* = 53)	*p* Value
*Demographics*			
Age, in years (SD)	68.2 (9.3)	66.8 (10.0)	0.440
Male gender (%)	39 (73.6)	38 (71.7)	0.828
*Quality of life modalities*			
Physical functioning	50.8 (32.8)	78.0 (23.0)	<0.001 *
Role limitations due to physical health	53.9 (34.0)	77.4 (25.8)	<0.001 *
Role limitations due to emotional problems	76.7 (29.4)	83.3 (25.5)	0.219
Energy/Fatigue	48.0 (21.8)	56.3 (20.1)	0.045 **
Emotional well-being	75.5 (15.9)	74.2 (16.7)	0.677
Social functioning	70.2 (30.5)	79.2 (25.9)	0.104
Pain	59.4 (27.9)	68.5 (27.3)	0.094
General Health	54.8 (23.6)	64.3 (20.1)	0.029 **
Health change	42.9 (19.8)	49.1 (18.3)	0.101

* *p* < 0.001; ** *p* < 0.05.

**Table 2:** Demographic, clinical, and quality-of-life-related characteristics of patients with gluten neuropathy reporting being on a gluten-free diet (GFD) compared to patients with gluten neuropathy reporting being on a gluten-containing diet.

	Gluten-Free Diet (*n* = 31)	Gluten-Containing Diet (*n* = 22)	*p* Value
*Demographics*			
Age, in years (SD)	68.6 (8.3)	67.6 (10.8)	0.702
Male gender (%)	20 (64.5)	19 (86.4)	0.115
*Clinical characteristics*			
Disease duration, in years (SD)	13.8 (8.0)	11.0 (10.2)	0.259
Type of neuropathy			0.690
Symmetrical length-dependent PN (%)	21 (67.8)	15 (68.2)	
Sensory ganglionopathy (%)	9 (29.0)	7 (31.8)	
Mononeuritis multiplex (%)	1 (3.2)	0 (0.0)	
Neuropathy severity			
Total ONLS score (SD)	3.1 (1.7)	3.2 (1.8)	0.814
*Quality of life modalities*			
Physical functioning	53.5 (34.3)	47.0 (31.0)	0.482
Role limitations due to physical health	56.0 (32.6)	50.9 (36.5)	0.589
Role limitations due to emotional problems	79.6 (25.2)	72.7 (34.7)	0.409
Energy/Fatigue	49.2 (23.5)	46.3 (19.5)	0.640
Emotional well-being	76.5 (16.1)	74.1 (15.9)	0.599
Social functioning	70.4 (27.4)	69.9 (35.1)	0.951
Pain	63.6 (28.0)	53.5 (27.3)	0.197
General Health	56.5 (22.9)	52.7 (24.9)	0.576
Health change	46.8 (19.1)	37.5 (20.0)	0.094

PN: peripheral neuropathy; ONLS: overall neuropathy limitations scale; SD: standard deviation.

**Table 3:** Demographic, clinical, and quality-of-life-related characteristics of patients with gluten neuropathy being on a strict gluten-free diet versus patients not being on a strict gluten-free diet (serologically confirmed by the elimination of anti-gliadin antibodies).

	On Strict GFD (*n* = 22)	Not on Strict GFD (*n* = 31)	*p* Value
*Demographics*			
Age, in years (SD)	69.7 (8.5)	67.2 (9.9)	0.329
Male gender (%)	14 (63.6)	25 (80.6)	0.166
*Clinical characteristics*			
Disease duration, in years (SD)	13.9 (8.1)	11.7 (9.6)	0.385
Type of neuropathy			0.462
Symmetrical length-dependent PN (%)	14 (63.6)	22 (71.0)	
Sensory ganglionopathy (%)	7 (31.8)	9 (29.0)	
Mononeuritis multiplex (%)	1 (4.5)	0 (0.0)	
Neuropathy severity			
Total ONLS score (SD)	3.2 (1.8)	3.0 (1.7)	0.695
*Quality of life modalities*			
Physical functioning	58.4 (35.7)	45.5 (30.0)	0.160
Role limitations due to physical health	58.5 (33.9)	50.6 (34.2)	0.409
Role limitations due to emotional problems	81.8 (23.7)	73.1 (32.8)	0.293
Energy/Fatigue	50.0 (26.1)	46.6 (18.5)	0.578
Emotional well-being	76.8 (18.2)	74.5 (14.2)	0.608
Social functioning	69.6 (28.4)	70.6 (32.4)	0.916
Pain	69.2 (26.6)	52.5 (27.1)	0.030*
General Health	56.4 (23.9)	53.9 (23.7)	0.708
Health change	48.9 (21.1)	38.7 (18.1)	0.066

GFD: gluten free diet; PN: peripheral neuropathy; ONLS: overall neuropathy limitations scale; SD: standard deviation.
